# Supplementary material for: Estimation of Children’s Soil and Dust Ingestion Rates and Health Risk at E-Waste Dismantling Area
Source: Int J Environ Res Public Health. 2022 Jun 15;19(12):7332. doi: 10.3390/ijerph19127332 (PMC9224165; doi:10.3390/ijerph19127332)
Supplement: Supplementary file 1 [file ijerph-19-07332-s001.zip › ijerph-1700078-supplementary.pdf]

Supporting Information

Estimation of Children's Soil and Dust Ingestion Rates and health risk in E-Waste Dismantling area

Yan Yang[a,b,c]*, Mengdi Zhang[a], Haojia Chen[a,b,c], Zenghua Qi[a], Chengcheng Liu[b], Qiang Chen[d], Tao Long[d]

[a]School of Environmental Science and Engineering, Institute of Environmental Health and Pollution Control, Guangdong University of Technology, Guangzhou, 510006, Guangdong, PR China
[b]Chemistry and Chemical Engineering Guangdong Laboratory, Shantou, 515041, Guangdong, PR China
[c]Synergy Innovation Institute of GDUT, Shantou, 515041, Guangdong, PR China
[d]State Environmental Protection Key Laboratory of Soil Environmental Management and Pollution Control, Nanjing Institute of Environmental Sciences, Ministry of Ecology and Environment of China, Nanjing, Jiangsu, China

*Corresponding author:

Yan Yang, Ph.D.

Rm 502, Engineering Facility Building No.3

Department of Environmental Science and Engineering

Guangdong University of Technology

Guangzhou, PR China

Tel: 020-39322564

Fax: 020-39322564

E-mail: yangyan1209@gdut.edu.cn

There are 10 pages including 4 figures and 6 tables.

■ Table of Contents

Items	Pages
**Figure S1.** Histogram and basic statistical parameters of investigated child population age, height, and weight.	Page S3
**Figure S2.** Histogram and basic statistical parameters of daily food ingestion (FOww) and feces (FCdw) and urine excretion for investigated child population.	Page S4
**Figure S3.** Histogram and basic statistical parameters of daily food ingestion (FOww) and feces (FCdw) and urine excretion for investigated child population.	Page S5
**Figure S4.** Frequency distribution histogram and outlier box of soil ingestion rate (SIR) based on tracer Al, Ba, Ce, Mn, Sc, Ti, V, and Y separately.	Page S6
**Table S1.** Basic statistical parameters of tracer element concentrations in food.	Page S7
**Table S2.** Basic statistical parameters of tracer element concentrations in faeces.	Page S8
**Table S3.** Basic statistical parameters of tracer element concentrations in urine.	Page S9
**Table S4.** Heavy metal concentration in soil of residential area.	Page S10
**Table S5.** Heavy metal concentration in soil of parks and green areas.	Page S11
**Table S6.** Value significance of carcinogenic risk and non-carcinogenic risk.	Page S12

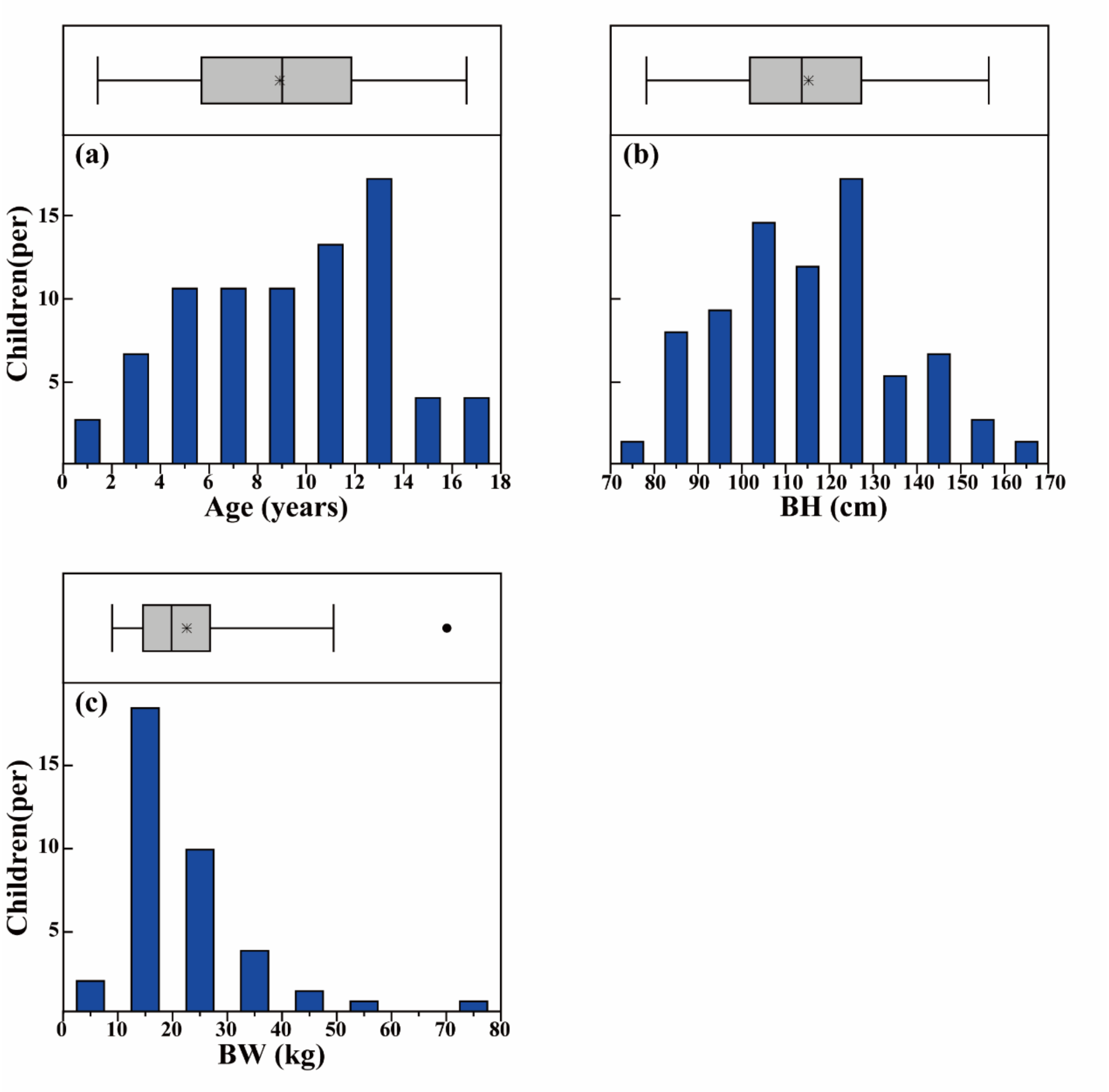

Figure S1. Histogram and basic statistical parameters of investigated child population age (**a**), height (**b**), and weight (**c**). The * represents mean values, and dot sign represents outliers.

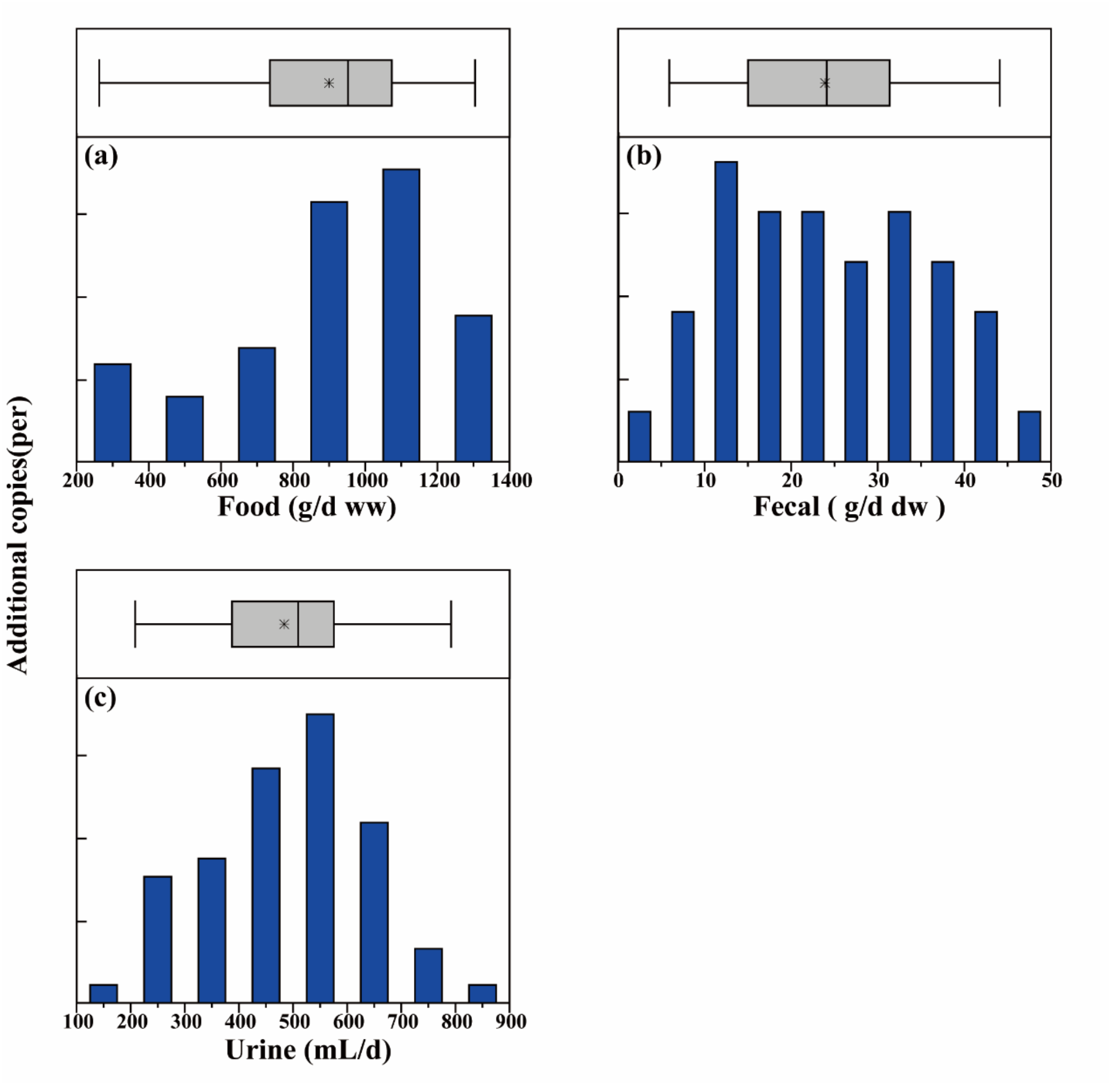

Figure S2. Histogram and basic statistical parameters of daily food ingestion (**a**) (g/d, ww) and feces (**b**) (g/d, dw) and urine (**c**) (mL/d) excretion for investigated child population. The * represents mean values.

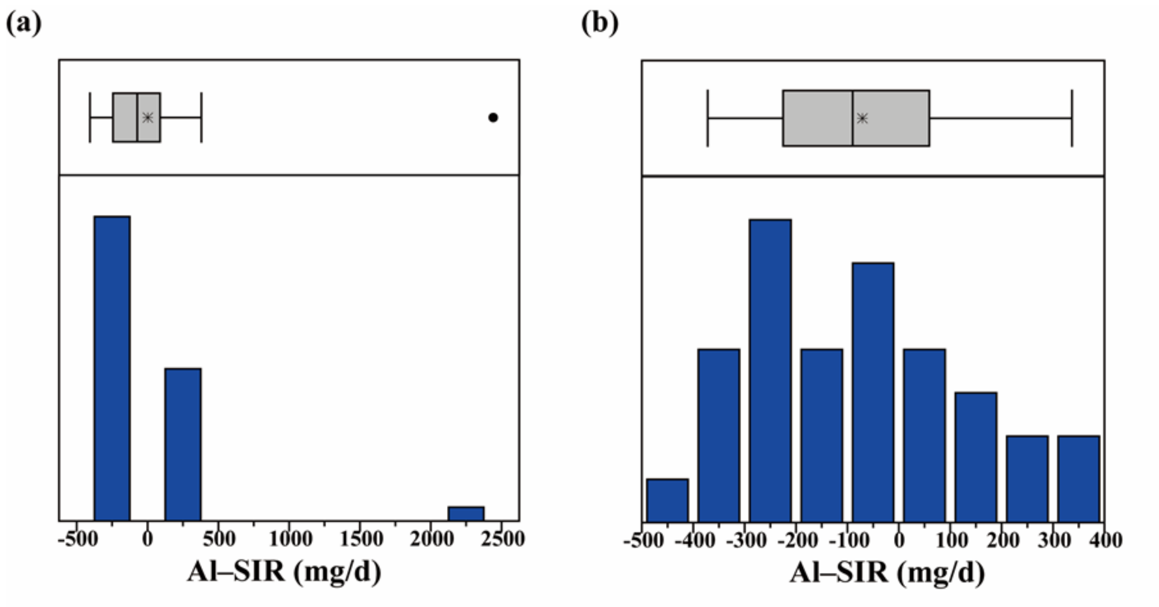

Figure S3. Frequency distribution histogram and outlier box (**a**) and Frequency distribution histogram and outlier box (**b**) of soil ingestion rate (SIR) based on Al. The * represents mean values, and dot sign represents outliers.

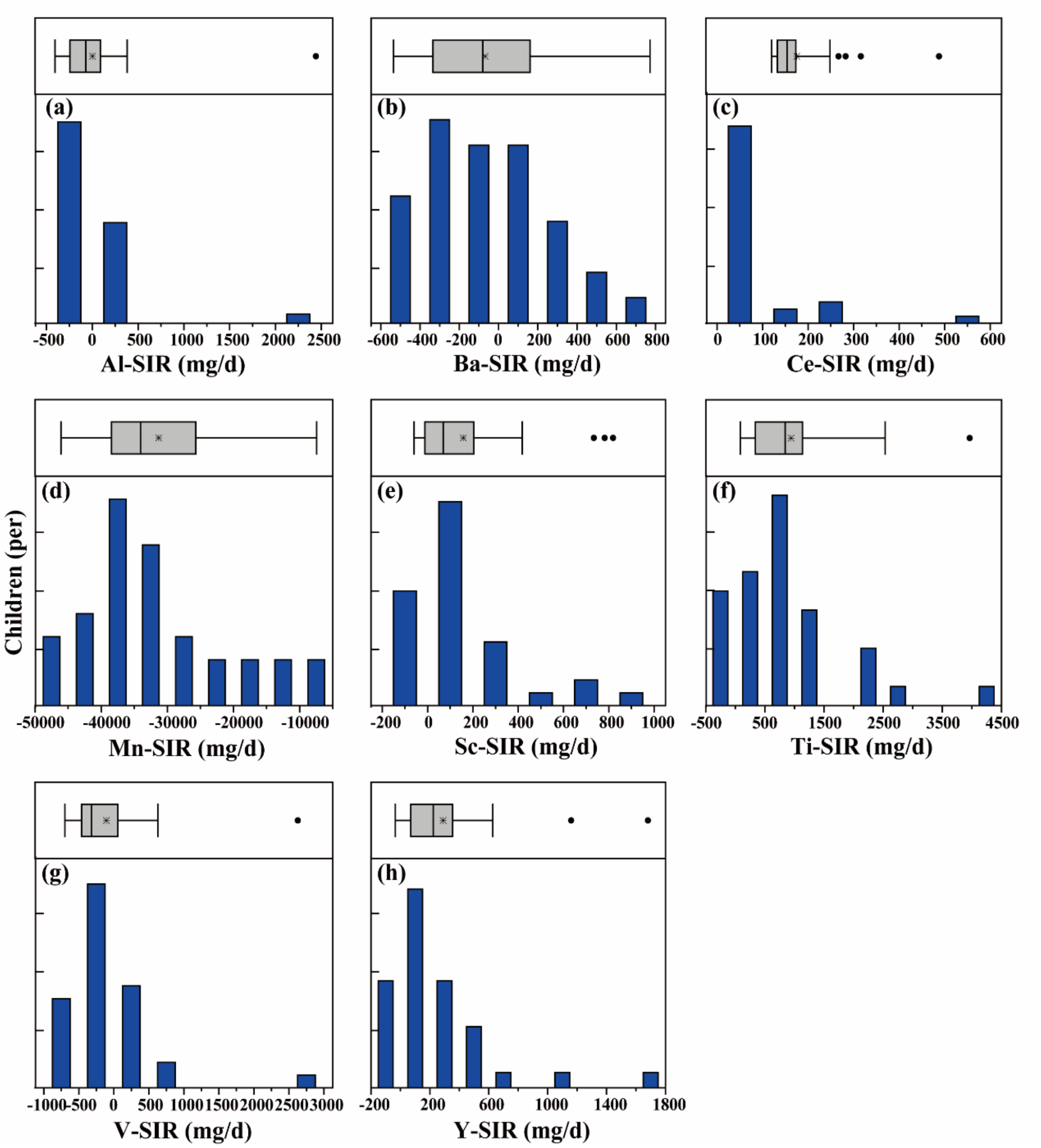

Figure S4. Frequency distribution histogram and outlier box of soil ingestion rate (SIR) based on tracer Al (**a**), Ba (**b**), Ce (**c**), Mn (**d**), Sc (**e**), Ti (**f**), V (**g**), and Y (**h**) separately. The * represents mean values, and dot sign represents outliers.

Table S1. Basic statistical parameters of tracer element concentrations in faeces.

	**Al**	**Ba**	**Ce**	**Mn**	**Sc**	**Ti**	**V**	**Y**
	**mg/kg**	**mg/kg**	**mg/kg**	**mg/kg**	**mg/kg**	**mg/kg**	**mg/kg**	**mg/kg**
Max	1487.32	11.03	1.08	73.67	0.46	548.73	2.04	0.76
99.5%	1479.59	10.97	1.07	70.30	0.43	463.33	2.01	0.68
97.5%	1426.15	10.73	1.04	57.35	0.34	141.74	1.90	0.37
95.0%	957.72	10.47	1.03	51.98	0.19	134.37	1.58	0.35
90.0%	742.83	9.55	0.82	50.49	0.12	113.56	1.14	0.29
75.0%	494.92	7.71	0.44	41.43	0.08	80.63	0.85	0.18
Median	265.74	5.28	0.25	31.04	0.05	55.54	0.64	0.12
25.0%	208.93	3.12	0.17	21.41	0.03	27.99	0.48	0.08
10.0%	139.29	1.74	0.09	7.68	0.01	21.53	0.36	0.06
2.5%	90.29	1.46	0.06	6.42	0.01	11.14	0.20	0.04
0.5%	114.97	1.47	0.07	6.57	0.01	15.70	0.31	0.04
Min	61.56	1.12	0.04	6.20	0.00	10.55	0.17	0.03
n	62	62	62	62	62	62	62	62

Table S2. Basic statistical parameters of tracer element concentrations in urine.

	**Al**	**Ba**	**Ce**	**Mn**	**Sc**	**Ti**	**V**	**Y**
	**mg/L**	**mg/L**	**μg/L**	**mg/L**	**μg/L**	**μg/L**	**μg/L**	**μg/L**
Max	900.00	0.00	1.30	0.00	1.10	181.00	6.90	0.00
99.5%	786.00	0.00	1.21	0.00	1.07	164.47	6.04	0.00
97.5%	457.50	0.00	0.64	0.00	0.91	122.58	3.86	0.00
95.0%	400.00	0.00	0.15	0.00	0.72	122.00	3.72	0.00
90.0%	100.00	0.00	0.15	0.00	0.15	2.50	0.25	0.00
75.0%	100.00	0.00	0.15	0.00	0.15	74.50	2.45	0.00
Median	100.00	0.00	0.15	0.00	0.15	45.00	1.15	0.00
25.0%	100.00	0.00	0.15	0.00	0.15	74.50	2.45	0.00
10.0%	100.00	0.00	0.15	0.00	0.15	17.70	0.25	0.00
2.5%	100.00	0.00	0.15	0.00	0.15	30.25	0.80	0.00
0.5%	100.00	0.00	0.15	0.00	0.15	5.21	0.25	0.00
Min	100.00	0.00	0.15	0.00	0.15	2.50	0.25	0.00
n	64	64	64	64	64	64	64	64

Table S3. Basic statistical parameters of tracer element concentrations in food.

	**Al**	**Ba**	**Ce**	**Mn**	**Sc**	**Ti**	**V**	**Y**
	**μg/kg**	**μg/kg**	**μg/kg**	**μg/kg**	**μg/kg**	**μg/kg**	**μg/kg**	**μg/kg**
Max	15500.00	490.00	0.50	12000.00	0.50	360.00	25.00	1.00
99.5%	15462.00	486.80	0.50	11998.00	0.50	359.30	25.00	1.00
97.5%	15310.00	474.00	0.50	11990.00	0.50	356.50	25.00	1.00
95.0%	15120.00	458.00	0.50	11980.00	0.50	353.00	25.00	1.00
90.0%	14740.00	426.00	0.50	11960.00	0.50	346.00	25.00	1.00
75.0%	13600.00	330.00	0.50	11900.00	0.50	325.00	25.00	1.00
Median	11700.00	170.00	0.50	11800.00	0.50	290.00	25.00	1.00
25.0%	10750.00	90.00	0.50	10850.00	0.50	285.00	16.00	1.00
10.0%	10180.00	42.00	0.50	10280.00	0.50	282.00	10.60	1.00
2.5%	9895.00	18.00	0.50	9995.00	0.50	280.50	7.90	1.00
0.5%	9819.00	11.60	0.50	9919.00	0.50	280.10	7.18	1.00
Min	9800.00	10.00	0.50	9900.00	0.50	280.00	7.00	1.00
n	66	66	66	66	66	66	66	66

Table S4. Heavy metal concentration in soil of residential area.

Sample	Cr	Ni	Cu	Zn	As	Cd	Pb
	mg/kg						
1	69.65	28.02	97.97	199.09	5.34	0.56	32.58
2	28.31	8.48	20.07	42.54	4.19	0.11	58.36
3	36.47	11.56	9.62	48.90	4.80	0.07	56.91
4	29.28	10.86	31.21	107.58	6.76	0.31	99.02
6	28.63	9.78	23.96	75.52	4.17	0.20	48.22
7	37.98	17.97	59.98	96.36	6.18	0.55	108.96
8	65.19	47.17	229.12	227.18	8.48	0.21	139.78
9	27.22	11.62	26.17	69.03	4.25	0.33	85.39
10	13.85	4.94	13.18	40.62	3.19	0.09	52.77
11	31.50	24.02	77.37	167.51	4.88	0.90	36.89
12	22.73	5.50	8.97	68.52	2.49	0.14	30.39
13	19.49	14.62	47.92	103.81	2.73	0.45	62.85
14	21.43	18.25	16.26	50.92	4.58	0.18	60.43
15	51.05	7.66	57.10	141.51	4.59	0.35	48.82
16	132.61	292.00	192.65	2714.06	17.67	0.66	191.19
17	90.91	239.93	171.89	1840.02	13.95	0.55	274.59
18	25.50	142.72	170.44	202.00	13.55	0.50	136.77
19	49.97	18.23	32.83	104.16	3.38	0.23	67.81
20	15.43	6.85	27.06	36.59	4.30	0.12	68.66
21	50.26	7.57	15.43	91.01	3.62	0.29	39.87
22	36.87	6.39	11.75	61.42	2.89	0.16	73.23
23	50.26	7.57	15.43	91.01	3.62	0.29	39.87
24	29.98	49.50	87.75	131.61	5.83	0.40	65.05

Table S5. Heavy metal concentration in soil of parks and green areas.

Sample	Cr	Ni	Cu	Zn	As	Cd	Pb
	mg/kg						
1	61.42	27.70	141.39	230.87	5.12	0.78	56.51
2	46.69	20.69	60.29	133.53	6.51	0.55	15.00
3	58.12	20.49	77.44	86.31	5.66	0.66	46.19
4	38.35	17.34	76.56	117.74	4.91	0.63	34.35
5	92.91	57.83	206.56	225.88	6.31	0.12	100.48
6	35.14	19.72	265.61	310.21	4.62	0.62	79.02
7	28.74	11.53	26.74	41.09	4.62	0.42	72.82
8	28.96	13.71	50.22	74.85	8.88	0.30	82.34
9	43.88	25.27	79.11	122.50	5.79	0.36	78.06
10	71.30	237.89	115.33	369.92	10.38	0.63	29.45
11	87.98	441.29	448.52	2908.13	13.09	1.40	388.54
12	69.49	176.36	117.02	1872.23	4.28	1.32	373.36
13	121.51	263.08	563.12	2401.51	12.10	1.36	536.61
14	15.81	7.51	28.90	63.38	2.55	0.20	62.24
15	28.43	9.62	58.56	88.21	3.59	0.29	43.88
16	55.84	8.11	14.10	64.50	2.51	0.13	42.92
17	69.24	22.03	162.77	320.70	4.35	0.87	45.66
18	36.87	6.39	11.75	61.42	2.89	0.16	73.23
19	23.98	48.30	189.83	176.45	2.76	0.39	35.52
20	50.26	7.57	15.43	91.01	3.62	0.29	39.87

Table S6. Value significance of carcinogenic risk and non-carcinogenic risk.

	**Not damage**	**Likely damage**	**Serious damage**
carcinogenic risk	$< 10^{6}$	$10^{6} < x < 10^{4}$	$10^{4} <$
non-carcinogenic risk (HQ)	< 1	$1 < x < 10$	$10 <$
